# Surgical Complications and Referral Patterns in 567 Patients with Differentiated Thyroid Cancer in the Northern Region of the Netherlands: A Population-Based Study Towards Clinical Management Implementation

**DOI:** 10.1245/s10434-020-08470-1

**Published:** 2020-04-23

**Authors:** Deborah van Dijk, Boukje A. C. van Dijk, Annemieke Weistra, Thera P. Links, John Th. M. Plukker

**Affiliations:** 1 Department of Surgical Oncology, University of Groningen, University Medical Center Groningen, Groningen, the Netherlands; 2 Department of Research and Development, Netherlands Comprehensive Cancer Organization (IKNL), Utrecht, the Netherlands; 3 Department of Epidemiology, University of Groningen, University Medical Center Groningen, Groningen, the Netherlands; 4 Department of Endocrinology, Internal Medicine, University of Groningen, University Medical Center Groningen, Groningen, the Netherlands

## Abstract

**Background:**

In the Netherlands, differentiated thyroid cancer (DTC) is treated surgically in three different hospital types, including university, teaching, and non- teaching peripheral hospitals. This study evaluates postoperative complications and referral patterns in patients with DTC in the northern region of the Netherlands to gain an understanding on how to improve management implementation.

**Methods:**

Data from 567 patients diagnosed between 1989 and 2009 were obtained from the Netherlands Cancer Registry and were supplemented with information from hospital digital information systems and patient records from 15 hospitals: 1 university, 3 teaching, and 11 peripheral hospitals. Surgically treated patients with a histologically proven DTC derived from the original pathology reports were included.

**Results:**

Surgical treatment could be performed in a single procedure in 234 patients (41.3%), but several surgeries were needed in the remaining 333 patients (58.7%). Recurrent laryngeal nerve (RLN) palsy occurred after all types of thyroid surgical procedures, but mostly after initial (hemi)thyroidectomy and reoperations. RLN was temporary in 3.2% of the nerves at risk and persistent in 1.8%. Temporary hypocalcemia developed in 13.7% of patients, and persistent hypocalcemia occurred in 4.8%. Patients were mainly referred to the university hospital from a non-teaching (40.7%, 48/118) or teaching hospital (11.1%, 16/144); however, 80% of patients were not referred.

**Conclusions:**

The complication rate and number of multiple surgeries support the efforts in optimizing clinical management in thyroid cancer. Careful considerations prior to initial surgical treatment by early discussion in telemedicine-based regional tumor boards could possibly prevent reoperations and potentially diminish complications.

**Electronic supplementary material:**

The online version of this article (10.1245/s10434-020-08470-1) contains supplementary material, which is available to authorized users.

The number of newly diagnosed patients with differentiated thyroid cancer (DTC) in the Netherlands has steadily increased from 250 in 1989 to 487 in 2009, and up to 607 in 2016.^1^ The rise in thyroid cancer incidence is likely due to the detection of early-stage tumors, and caused by overdiagnosis.^2^^–^4 Generally, DTC is a well-treatable disease with a favorable prognosis and a 10-year survival rate of 80–95%.^5^,^6^ Surgery is the basis of treatment with curative intent for patients with DTC, but has potentially harmful consequences. The most common complications after surgical treatment of thyroid cancer are hypoparathyroidism and recurrent laryngeal nerve (RLN) paralysis, with reported risks of 1–38% and 0.9–8.0%,^7^^–^12 respectively, both leading to lifelong morbidity and diminished quality of life (QoL).^13^ With the increasing incidence of low-risk DTC in the last decades, the extent of surgery has been reduced, thereby preventing surgical complications, while cancer-related survival remained equal.^14^

Morbidity in DTC patients can be reduced by accurate selection of patients and optimizing treatment strategies by standardizing treatment protocols and referral patterns. Traditionally, patients with DTC in the Netherlands were referred for surgical treatment based on expert opinion, surgeon experience, extensiveness of the disease, and the extent of primary or secondary treatment. The National Thyroid Cancer treatment guidelines update was published in 2007 to ensure that treatment decisions are made in a consistent manner across all hospitals for every patient with a suspected thyroid nodule or DTC.^15^ Based on these guidelines, treatment protocols were gradually standardized throughout the country, aiming to improve adequate initial treatment strategies with less reoperations and thereby decreasing morbidity through better QoL in patients with DTC.

Guidelines for the treatment of thyroid cancer are largely established on expert opinion and/or are consensus-based. Even today, most data are retrieved from retrospective studies, as the possibility of prospective studies remains limited in these patients. Validation studies are important to assess patient outcomes after implementation of current guidelines and to understand the impact of modifications in the guidelines for treatment of DTC; therefore, these large, population-based studies are still indispensable. They can be used as validation and for future reference in comparing outcomes after prospective guideline modifications.

In this study, we evaluated surgical complications in DTC patients, in particular RLN palsy and hypocalcemia. We correlated these to the extent and number of surgical procedures for DTC with respect to hospital level and referral patterns in the northern part of the Netherlands between 1989 and 2009, i.e. the period before consensus management and full implementation of the Dutch Thyroid Guidelines.

## Methods

### Patient and Data Collection

The northern region of the Netherlands, a catchment area for 2.2 million patients, harbors 1 university medical center, 3 teaching hospitals, and 12 non-teaching, peripheral hospitals.^16^ From 1989 on, data from all DTC patients treated in the Netherlands have been registered by the Netherlands Cancer Registry (NCR). Population-based data retrieved from the NCR are based on notification of all newly diagnosed malignancies in the Netherlands by the Pathological Anatomical National Automated Archive (PALGA), supplemented by information from the national registry of cancer-related hospital discharges. Completeness is estimated at > 95%.^17^ In this retrospective study, we identified 791 DTC patients in the NCR who were treated in the northern region between 1989 and 2009, prior to consensus management and full implementation of the Dutch Thyroid Guidelines.

Fifteen of the 16 hospitals agreed to participate and approved additional data collection for this study. One small non-teaching peripheral hospital declined our request to collect additional patient information, leaving 11 peripheral hospitals with a total of 780 patients. Patients were included when the original pathology reports of thyroid surgical procedures could be retrieved. For 192 patients, pathology reports were not available. Patients who did not receive any surgical treatment, e.g. patients with tumors found at autopsy (*n* = 18) and patients who died before treatment could be initiated (*n* = 3) were excluded for all calculations, resulting in a total of 567 included patients. Demographics of the included and excluded patients are provided in electronic supplementary Table S1.

Data from the NCR of all included patients were uniformly supplemented with data from digital hospital information systems and patient records. All well-differentiated thyroid cancer variants (papillary, follicular, and Hürthle cell carcinomas) were included. Histology was registered according to the International Classification of Diseases for Oncology, Third Edition (ICD-O-3). During the study period, we used the concurrent version of the TNM classification system, including the 3rd edition from 1989 to 1992, the 4th edition between 1993 and 1997, the 5th edition between 1998 and 2002, and the 6th edition from 2003 to 2009. Because of the retrospective design of this population-based study and the anonymous data processing, the Institutional Review Board waived further approval according to Dutch law at the time of inclusion. All data were fully anonymized before performing analyses.

### Definitions

Recurrent or persistent disease is usually an indication for follow-up surgery. Recurrence was defined as detectable Tg-on (i.e. Tg during substitution therapy) and/or tumor detectable, either on diagnostic imaging or at physical examination, commonly with cytological or histological confirmation of tumor during follow-up after an initial curation.^18^

Persistent disease was defined as a continuous existence of microscopic disease (i.e. cytopathologically/histopathologically and/or detectable Tg-on) or detectable macroscopic disease (i.e. on physical examination, imaging, or last surgery), as previously described.^18^,^19^

Transient or persistent RLN paralysis was defined as paralysis of one or both RLNs for a period of < 1 year or > 1 year, respectively. Nerve dysfunction was diagnosed either peroperatively, clinically, or through laryngoscopy. Intentionally sacrificed nerves due to gross tumor invasion or encasement, visible during surgery, were not recorded as a complication. Transient and persistent hypocalcemia was defined as low serum calcium, for which suppletion therapy with calcium and/or active vitamin D was necessary for < 1 year or > 1 year, respectively. TNM stage was based on the original pathology report after initial surgery and, if applicable, on the results of the first post-ablation radioactive iodine (^131^I) scan.

### Subdivision of Hospitals

To assess treatment strategies and referral patterns, the participating hospitals were subdivided into three categories as described in the Dutch 2007 guidelines: Level IA: university hospital; Level IB: teaching hospital; and Level II: non-teaching, peripheral hospital.^15^ These levels were implemented after the inclusion period of this study.

### Subdivision of Surgical Procedures

In order to classify the surgically related complications, operations were categorized according to the anatomically defined surgical procedures described by van Dijk et al.^20^ and Robbins et al.^21^ (Fig. 2).

1. Surgery in the central compartment (i.e. thyroid region and nodes in levels VI and VII):
  HT ± CCD/LND: initial hemithyroidectomy (HT; with or without unilateral central compartment lymph node [LN] dissection [CCD]; or HT with additional exploration/LN dissection in the lateral neck [LND]).
  TT ± CCD: total thyroidectomy (TT; with or without central compartment LN dissection [CCD]).
  TT ± CCD + LND: total thyroidectomy with additional lateral neck dissection (with or without central compartment LN dissection).
  MISC: miscellaneous (surgery not otherwise specified; mediastinoscopy with node removal; metastasectomy; neck exploration).
  REOP CCD UNIL/BIL: reoperation in the central compartment, unilateral/bilateral.
2. Surgery only in the lateral compartment (i.e. LN dissection at levels II–V):
  UNIL LND: unilateral lateral neck dissection.
  BIL LND: bilateral lateral neck dissection.
  REOP LND: reoperation outside the central compartment (lateral neck dissection).

### Surgical-Related Complications

Surgical complications were scored per surgical procedure. Some patients had multiple complications after one surgical procedure. To determine RLN paralysis, it is important to know whether patients were operated unilaterally or bilaterally, since one or both laryngeal nerves could be at risk, respectively. Therefore, RLN paralysis was scored per nerve at risk. Thus, bilateral surgical procedures (type TT ± CCD, TT ± CCD + LND, and REOP BIL) are multiplied by a factor of two (Table 4).

### Statistical Methods

Normally distributed data are expressed as mean with standard deviation (SD). Numbers and percentages are reported throughout the paper. Chi square or Fisher exact tests were used when applicable. A two-sided *p* value < 0.05 was considered to be statistically significant. Statistical analyses were performed using IBM SPSS for Windows version 23 (IBM Corporation, Armonk, NY, USA).

## Results

### Patient and Tumor Characteristics

**Fig. 1:**
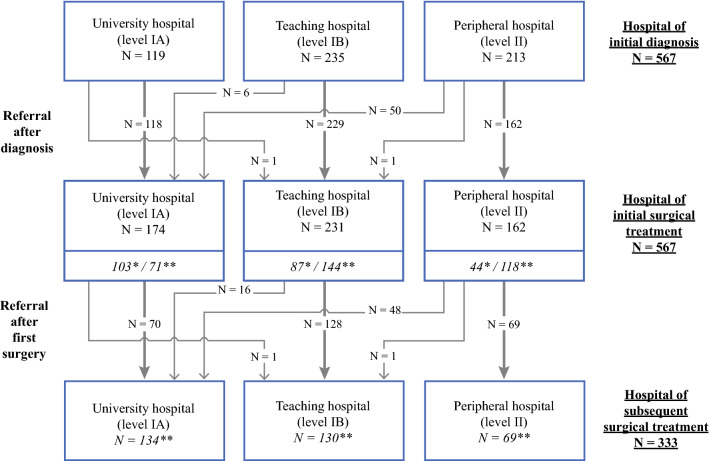
Referral patterns in the northern part of the Netherlands between 1989 and 2009. Of the total 567 patients, 58 were immediately referred, after initial diagnosis, to another hospital for surgical treatment. *A total of 234 (103 + 87 + 44) patients (41.3%) received one operation. **A total of 333 (71 + 144 + 118) patients (58.7%) received multiple surgical treatments

Patient and tumor characteristics of the 567 included patients, at the time of diagnosis, are reported in Table 1. Most patients had a papillary thyroid carcinoma (*n* = 419, 73.9%) and were diagnosed at a mean age of 49.2 years (SD 17.7). The majority had a T1 or T2 tumor (*n* = 352, 62.1%), whereas 195 patients (34.4%) presented with a T3 or T4 locally advanced thyroid carcinoma; 159 (28.0%) patients had LN metastases at presentation and 26 (4.6%) presented with distant metastases. DTC was an incidental finding during non-thyroid-related surgery in the head and neck region in 30 patients (5.3%); In most of these cases, a median or lateral neck cyst or reactive LN was suspected. If these 30 patients underwent subsequent hemithyroidectomy or total thyroidectomy with or without additional central compartment LN dissection, these surgical interventions were scored as the ‘initial thyroid cancer-related operation’. The included 567 patients underwent 948 thyroid-related surgical procedures (Table 1). In total, 234 patients (41.3%) had one operation, whereas the remaining 333 patients (58.7%) underwent multiple surgeries (Fig. 1). Of these 333 patients, 296 (52.2%) underwent two operations, 28 (4.9%) underwent three operations, 7 (1.2%) underwent four operations, and 2 (0.4%) patients underwent five operations. Patients with follicular thyroid carcinoma more often had multiple surgeries compared with patients with papillary thyroid carcinoma (*p* < 0.01); 76.4% of patients with follicular thyroid carcinoma received multiple operations, versus 52.5% of patients with papillary carcinoma. Patients with locally advanced T3 or T4 tumors were significantly more often treated with multiple surgical procedures than patients with smaller tumors (*p* = 0.02) (Table 1); they were more frequently operated in the university hospital (41.1%) compared with teaching (32.6%) and peripheral hospitals (29.6%), although this was not significantly different (*p* = 0.06).

**Table 1:** Patient and tumor characteristics at diagnosis, and related numbers of surgery

	Patients	Surgeries	One surgery	Multiple surgeries
Total	567 (100)	948 (100)	234 (100)	333 (100)
Male	150 (26.5)	260 (27.4)	60 (25.6)	90 (27.0)
Female	417 (73.5)	688 (72.6)	174 (74.4)	243 (73.0)
Age at diagnosis, years (mean ± SD)	49.2 ± 17.7	NA	51.6 ± 19.2	47.6 ± 16.4
Papillary carcinoma	419 (73.9)	673 (71.0)	199 (85.1)	220 (66.1)
Follicular carcinoma (including Hürthle)	148 (26.1)	275 (29.0)	35 (14.9)	113 (33.9)^a^
TX	20 (3.5)	37 (3.9)	5 (2.1)	15 (4.5)
T1	139 (24.5)	209 (22.0)	75 (32.1)	64 (19.2)
T2	213 (37.6)	361 (38.1)	75 (32.1)	138 (41.5)
T3	112 (19.8)	192 (20.3)	44 (18.8)	68 (20.4)
T4	83 (14.6)	149 (15.7)	35 (15.0)	48 (14.4)
NX	319 (56.3)	544 (57.4)	112 (47.9)	207 (62.2)
N0	89 (15.7)	140 (14.8)	40 (17.1)	49 (14.7)
N1	159 (28.0)	264 (27.8)	82 (35.0)	77 (23.1)
MX	165 (29.1)	278 (29.3)	70 (29.9)	95 (28.5)
M0	376 (66.3)	625 (65.9)	154 (65.8)	222 (66.7)
M1	26 (4.6)	45 (4.8)	10 (4.3)	16 (4.8)

Data are expressed as *n* (%) unless otherwise specified

*T* tumor, *N* nodes, *M* metastases, *X* unknown, *NA* not applicable, *SD* standard deviation

^a^Patients with follicular thyroid carcinoma more often had multiple surgeries compared with patients with papillary thyroid carcinoma (*p* < 0.01)

### Referral Patterns

Referral patterns of DTC patients in the northern part of the Netherlands are shown in Fig. 1. Fifty-eight of 567 patients (10.2%) were directly referred, after initial diagnosis, to the university hospital (*n* = 56, 96.6%) or teaching hospital (*n* = 2, 3.4%) for surgical treatment. In these 58 patients, locally advanced tumors (T3/T4) were present in 28 patients (47.5%), LN metastases were present in 35 patients (55.9%), and distant metastases were present in 9 patients (15.3%).

Of the 234 patients with one surgical procedure, 103 (44.0%, 103/234) were operated in the university hospital, 87 (37.0%, 87/234) were operated in the teaching hospitals, and 44 (18.8%, 44/234) were operated in the peripheral hospitals. Of the 174 patients who had their initial operation in the university hospital, 40.8% (71/174) had a subsequent resection, compared with 62.3% (144/231) and 72.8% (118/162) in the teaching and peripheral hospitals, respectively (*p* < 0.01).

If a second operation was needed, 80% of patients were treated in the hospital of the primary surgery (267/333, 80.2%). If referral took place, patients were mostly referred from a peripheral (40.7%, 48/118) or teaching hospital (11.1%, 16/144) to the university hospital. There was no difference in referral rate between patients with locally advanced tumors (T3/T4; 40.8%) and patients with small tumors (T1/T2; 59.2%) [*p* = 0.24]. There was no difference in complication rate in referred (46.9%, 23/49) versus unreferred (31.9%, 22/69) patients treated initially in a peripheral hospital (*p* = 0.23).

Two patients were referred from the university hospital to a teaching hospital—one after initial diagnosis and one for follow-up surgery. No patients were referred from the university and teaching hospitals to peripheral hospitals. Of the 134 patients who had their subsequent surgery in the university hospital, 64 (47.8%) were referred, of whom 75.0% (48/64) were referred from peripheral hospitals.

### Surgical Procedures Regarding Hospital Level

**Fig. 2:**
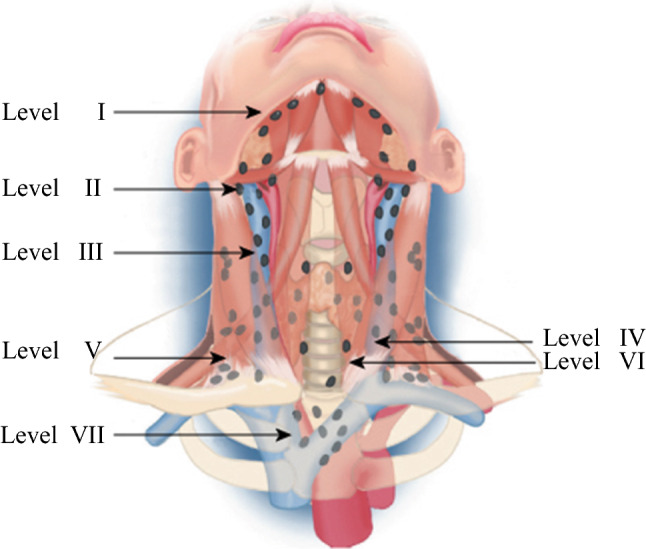
The system of lymph node levels in the neck, as described by Robbins et al.^21^ Level I: submental and submandibular group, lymph nodes within the boundary of the subdigastric muscles and the hyoid bone. Levels II/III/IV: Upper, middle, and lower jugular group, lymph nodes located around the internal jugular vein, sternohyoid muscle anteriorly, sternocleidomastoid muscle posteriorly, skull base superiorly, and clavicle inferiorly. Level V: Posterior triangle group, located between the sternocleidomastoid muscle and the trapezius muscle, including suprasternal lymph nodes. Level VI: Anterior compartment, located in the midline between the carotid sheets, from the hyoid bone superiorly to the suprasternal notch inferiorly. Level VII: Mediastinal lymph nodes. Image modified from de Groot et al.^20^

As shown in Tables 2 and 3, all surgical procedures were subdivided according to their localization in the neck, i.e. at the central (Table 2) or lateral compartment (Table 3, Fig. 2), and hospital level. In the teaching and peripheral hospitals, hemithyroidectomy as an initial procedure was most often performed in 37.6% (137/364) and 52.4. % (122/233) of patients, respectively. By comparison, in the university hospital, total thyroidectomy (with or without lateral compartment dissection) was more often performed as initial surgery in 44.4% (136/306) of patients.

**Table 2:** Total numbers of surgery within the central compartment according to hospital level

Type of surgery	University hospital	Teaching hospital	Peripheral hospital	Total
HT ± CCD/LND	47 (15.4)	137 (44.8)	122 (39.8)	306 (100)
TT ± CCD	79 (50.3)	56 (35.7)	22 (14.0)	157 (100)
TT ± CCD + LND	57 (69.5)	24 (29.3)	1 (1.2)	82 (100)
MISC	40 (45.5)	29 (33.0)	19 (21.5)	88 (100)
REOP CCD UNIL/BIL	83 (30.7)	118 (43.7)	69 (25.6)	270 (100)
Total	306 (33.9)	364 (40.3)	233 (25.8)	903 (100)

Data are expressed as *n* (%)

Operations including both the central and lateral compartments are listed in Tables 2 and 3

*HT* ± *CCD/LND* hemithyroidectomy (with or without central or lateral compartment LN dissection), *TT* ± *CCD* total thyroidectomy (with or without central compartment LN dissection), *TT* ± *CCD* + *LND* total thyroidectomy with lateral compartment LN dissection (with or without central compartment LN dissection), *MISC* other (surgery not otherwise specified, mediastinoscopy, metastasectomy), *REOP CCD UNIL/BIL*: reoperation in the central compartment, unilateral or bilateral, *LN* lymph node

**Table 3:** Total numbers of surgery limited to the lateral compartment according to hospital level

Type of LN dissection	University hospital	Teaching hospital	Peripheral hospital	Total
UNIL LND	29 (56.9)	18 (35.3)	4 (7.8)	51 (100)
BIL LND	7 (70.0)	3 (30.0)	0 (0.0)	10 (100)
REOP LND	25 (89.3)	3 (10.7)	0 (0.0)	28 (100)
Total	61 (68.5)	24 (27.0)	4 (4.5)	89 (100)

Data are expressed as *n* (%)

Operations including both the central and lateral compartments are listed in Tables 2 and Table 3

*UNIL LND* unilateral LN dissection, *BIL LND* bilateral LN dissection, *REOP LND* reoperation LN dissection outside the central compartment, *LN* lymph node

Lateral neck dissections, whether unilateral or bilateral, were commonly performed in the university and teaching hospitals (Table 3). A unilateral lateral neck dissection was carried out in 29 patients (56.9%) in the university hospital, 18 patients (35.3%) in the teaching hospitals, and 4 patients (7.8%) in the peripheral hospitals. Bilateral lateral neck dissections and reoperations of nodal metastases in the lateral neck (levels II–VI) were mostly (70.0% and 89.3%, respectively) performed in the university hospital, while 30.0% and 10.7%, respectively, were performed in teaching hospitals.

### Thyroid Surgery-Specific Complications

The two most common thyroid surgery-specific complications—RLN palsy and hypocalcemia—are outlined in Tables 4 and 5. Table 4 shows the percentage of RLN palsy according to the type of surgery in the central compartment. In 3.2% of the nerves at risk, a temporary RLN palsy occurred and RLN palsy was persistent in 1.8%. RLN palsy occurred after all types of surgery, i.e. in 19 patients (6.2%) after initial hemithyroidectomy, 16 patients (5.1%) after total thyroidectomy, and 13 patients (4.5%) after reoperation. Nine patients (16.1%, 9/56) operated in the university hospital developed RLN palsy, versus 25 (44.6%, 25/56) patients operated in the teaching hospitals and 22 (39.3%, 22/56) in the peripheral hospitals (*p* = 0.9).

**Table 4:** Recurrent nerve palsy according to type of surgery and nerves at risk

Type of surgery	Total number of nerves at risk	Recurrent nerve palsy [*n* (%)]		
		Temporary	Persistent	Total
HT ± CCD/LND	306	12 (3.9)	7 (2.3)	19 (6.2)
TT ± CCD	314	10 (3.2)	6 (1.9)	16 (5.1)
TT ± CCD + LND	164	5 (3.1)	1 (0.6)	6 (3.7)
MISC	69	0 (0.0)	1 (1.5)	1 (1.5)
REOP CCD UNIL/BIL	289	9 (3.1)	4 (1.4)	13 (4.5)
Total	1142	36 (3.2)	20 (1.8)^a^	56 (4.9)

*HT* ± *CCD/LND* hemithyroidectomy (with or without central or lateral compartment dissection), *TT* ± *CCD* total thyroidectomy (with or without central compartment dissection), *TT* ± *CCD* + *LND t*otal thyroidectomy with lateral compartment dissection (with or without central compartment dissection), *MISC* other (surgery not otherwise specified, mediastinoscopy, metastasectomy), *REOP CCD UNIL/BIL* reoperation in the central compartment, unilateral or bilateral. Bilateral surgeries are included for each side

^a^One patient developed persistent recurrent nerve palsy after lateral neck dissection, following initial surgery, in the central compartment

**Table 5:** Hypocalcemia according to type of surgery and number of patients

Type of surgery	Total number of patients	Hypocalcemia [*n* (%)]		
		Temporary (+ unknown)	Permanent	Total
HT ± CCD/LND	306	9 (0.3)	2 (0.006)	11 (0.04)
TT ± CCD	157	32 (20.4)	11 (7.0)	43 (27.3)
TT ± CCD + LND	82	30 (36.6)	14 (17.1)	44 (53.7)
MISC	88	3 (3.4)	0 (0.0)	3 (3.4)
REOP CCD UNIL/BIL	270	49 (18.2)	16 (5.9)	65 (24.1)
Total	903	124 (13.7)^a^	43 (4.8)	167 (18.5)

HT ± CCD/LND hemithyroidectomy (with or without central or lateral compartment dissection), *TT* ± *CCD* total thyroidectomy (with or without central compartment dissection), *TT* ± *CCD* + *LND* total thyroidectomy with lateral compartment dissection (with or without central compartment dissection), *MISC* other (surgery not otherwise specified, mediastinoscopy, metastasectomy), *REOP CCD UNIL/BIL* reoperation in the central compartment, unilateral or bilateral

^a^One patient developed temporary hypocalcemia after reoperation in the lateral compartment following extensive surgery, in the central and lateral compartment

In Table 5, the percentages of postoperative temporary and persistent hypocalcemia are shown according to the type of surgery in the central compartment. In some patients, the exact duration of recurrent nerve palsy (*n* = 4/1142 total nerves at risk) or hypocalcemia (*n* = 30/903 surgeries with postoperative hypocalcemia) was unknown. These patients were categorized as transient for the analyses. In total, 124 patients (13.7%) developed temporary hypocalcemia and 43 patients (4.8%) had persistent hypocalcemia. Hypocalcemia occurred most frequently after initial total thyroidectomy with (*n* = 44, 53.7%) or without (*n* = 43, 27.3%) a concomitant lateral neck dissection, and in 65 patients (24.1%) after reoperation. Nine patients developed temporary hypocalcemia after an initial hemithyroidectomy, of whom two patients even developed permanent hypocalcemia.

Other thyroid surgery-related complications (electronic supplementary Table S2) were Horner’s syndrome in two patients, a partial facial nerve palsy in two patients, and an accessory nerve palsy in four patients. One patient had a vagal nerve palsy and two patients experienced a temporary hypoglossal nerve palsy. Chyle leakage was reported in seven patients.

### Other Complications

General postoperative complications, recorded up to 1 year after surgery, included postoperative bleeding in 33 patients, wound infection in 22 patients, and pneumonia in 9 patients (electronic supplementary Table S2).

## Discussion

This retrospective study gives an overview of thyroid cancer-specific complications in DTC patients in regard to the type and number of surgeries related to hospital level and referral pattern in the northern part of the Netherlands between 1989 and 2009. Our data show that all types of thyroid cancer surgery were performed in all hospital levels, which may lead to increased thyroid surgery-related complications. Patients with follicular thyroid carcinoma more often had multiple surgeries compared with patients with papillary thyroid carcinoma. Higher T-stage tumors require a more extensive and higher number of operative treatments, which is more or less biological inherent. Our data further show that patients treated in the university hospital have less follow-up surgeries compared with the other hospital levels. Since high complication rates were seen after reoperation, we advocate early discussion in regional tumor boards prior to initial treatment to prevent follow-up surgeries and potentially prevent complications.

In this cohort, temporary RLN palsy occurred in approximately 3% of the nerves at risk, and persistent RLN palsy occurred in < 2% (Table 4). These rates are in accordance with the literature, with temporary and persistent RLN palsy rates of between 2.0 and 8.0%, and 0.9 and 3.0%, respectively.^11^,^22^ High percentages of RLN palsy were seen in the reoperation group, but even higher percentages were seen in the initial (hemi)thyroidectomy group. This might be explained by the fact that hemithyroidectomies were performed in all hospitals, including low-volume hospitals. Higher percentages of RLN palsy were seen in the teaching and peripheral hospitals, although, due to small numbers, this was not statistically different. An association between a lower risk of RLN palsy in a high-volume center has been found by others,^23^^–^26 and is also in accordance with a previous study in which a better outcome was suggested in patients with rare tumors when treated in a high-volume center.^27^

Temporary hypocalcemia was observed in about 14% of DTC patients after thyroid surgery, and persistent hypocalcemia was observed in almost 5%. These results are comparable with earlier reports, with temporary hypocalcemia reported at 8% and 38%, and persistent hypocalcemia at 1% and 3%, respectively.^12^,^28^^–^30 Next to initial total thyroidectomy, with or without concomitant lateral compartment LN dissection, the highest percentage of hypocalcemia was seen after reoperative surgery. As described above, the type of thyroid surgery causing RLN palsy was also at high risk of causing hypocalcemia. Our data show that these procedures were performed in all hospitals, including low-volume centers, during the period of the study. However, note that our data only include malignant thyroid surgery; benign surgery may have been performed more frequently in hospitals with low-volume thyroid cancer surgeries.

Our results also show that the majority of patients were not referred to another (different level) hospital in case of multiple operations. In addition, there was no difference in referral patterns between patients with locally advanced (T3-T4) tumors and small (T1-T2) tumors.

Early referral should especially be considered in patients with locally advanced (T3–4) tumors, since these patients frequently present with extrathyroidal spread and older age (> 50 years), with a concomitant higher risk of incomplete resection.^31^ In accordance with the literature, 75% of the T3–4 tumors in our study were treated in high-volume hospitals as they require more extensive surgical resection or reoperations. However, we should be aware of the preoperative signs, as almost one-quarter of patients with T3 or T4 tumors (*n* = 48, 24.6%) still had their primary surgical resection outside the university hospital. If in doubt regarding extrathyroidal extension, proper preoperative evaluation, including at least magnetic resonance imaging and ultrasonography of the cervical region with laryngeal examination, is recommended. These requirements are not only needed to prevent reoperations but also for good judgment in difficult cases during surgery, including attempts to preserve the RLN, even in case of partial encasement.

In 2007, the first national Dutch Thyroid Guidelines were completed and gradually introduced into all hospitals up to 2009 and thereafter.^15^ The data reported in this study, including thyroid-specific, surgical-related complications, should be seen as supporting data for the proposed workflow and hospital classification by level. These data can be used as baseline data for comparing treatment outcomes and QoL in future studies. Moreover these results will offer us more insight into whether complication rates are diminishing as a result of the current trend towards a less aggressive treatment approach, with less extensive surgery and even abstention of treatment in low-risk patients, as suggested in the most recent American Thyroid Association (ATA) guidelines.^14^,^32^^–^36

Due to the retrospective design of this study, there are a number of limitations. We had to extract data on patient, tumor, and treatment characteristics from patient records and are therefore dependent on data recorded at that time. Inclusion bias may have occurred, since more data were available in the later years of the inclusion period, and therefore more patients had to be excluded in the early inclusion period (electronic supplementary Table 1). Moreover, our exclusion group contained more follicular thyroid carcinomas, more T1 stage tumors, and more M0 stage tumors, which could lead to bias since this group might have more favorable characteristics than our inclusion group. Pathology reports were standardized only in the later inclusion years of this study, and therefore a number of TNM stages are missing. Standardizing complication registration occurred more frequently and earlier in the university hospital, which could have led to a relative underreporting of complications from the teaching and peripheral hospitals. Moreover, preoperative laryngoscopy was not routinely performed, which may have led to a relative overreporting of RLN palsy, especially in patients who only underwent a hemithyroidectomy.

## Conclusions

The results of this population-based study, showing highest RLN and hypocalcemia complication rates after initial (hemi)thyroidectomy (with or without lateral compartment dissection) and after reoperations, support the efforts in optimizing clinical management in thyroid cancer. Careful consideration should be made prior to initial surgical treatment by way of early discussion in telemedicine-based regional tumor boards. This may lead to better selection of low- and high-risk patients, with more patient-oriented treatment of differentiated thyroid carcinoma, as has been introduced in more recent years.

## Electronic supplementary material

Below is the link to the electronic supplementary material.

Supplementary material 1 (DOCX 13 kb)

## References

[1] Table incidence thyroid cancer 1989–2019, Netherlands Cancer Registry. Available at: https://www.cijfersoverkanker.nl/selecties/dataset_1/img5b34a7d166418. Accessed 28 Oct 2019.

[2] Welch HG, Black WC (2010). Overdiagnosis in cancer. J Natl Cancer Inst..

[3] Davies L, Welch HG (2006). Increasing incidence of thyroid cancer in the United States, 1973-2002. JAMA..

[4] Ahn HS, Kim HJ, Welch HG (2014). Korea’s thyroid-cancer “epidemic”: screening and overdiagnosis. N Engl J Med..

[5] Bilimoria KY, Bentrem DJ, Ko CY, et al. Extent of surgery affects survival for papillary thyroid cancer. *Ann Surg*. 2007;246(3):375–81 **(discussion 381–4)**. 10.1097/SLA.0b013e31814697d9 PMC1959355 17717441

[6] Kelly A, Barres B, Kwiatkowski F (2019). Age, thyroglobulin levels and ATA risk stratification predict 10-year survival rate of differentiated thyroid cancer patients. PLoS One..

[7] Cheah WK, Arici C, Ituarte PH, Siperstein AE, Duh QY, Clark OH (2002). Complications of neck dissection for thyroid cancer. World J Surg..

[8] Chisholm EJ, Kulinskaya E, Tolley NS (2009). Systematic review and meta-analysis of the adverse effects of thyroidectomy combined with central neck dissection as compared with thyroidectomy alone. Laryngoscope..

[9] Giordano D, Valcavi R, Thompson GB (2012). Complications of central neck dissection in patients with papillary thyroid carcinoma: results of a study on 1087 patients and review of the literature. Thyroid..

[10] Glockzin G, Hornung M, Kienle K (2012). Completion thyroidectomy: effect of timing on clinical complications and oncologic outcome in patients with differentiated thyroid cancer. World J Surg..

[11] Pisanu A, Porceddu G, Podda M, Cois A, Uccheddu A (2014). Systematic review with meta-analysis of studies comparing intraoperative neuromonitoring of recurrent laryngeal nerves versus visualization alone during thyroidectomy. J Surg Res..

[12] Rosato L, Avenia N, Bernante P (2004). Complications of thyroid surgery: analysis of a multicentric study on 14,934 patients operated on in Italy over 5 years. World J Surg..

[13] Husson O, Haak HR, Buffart LM (2013). Health-related quality of life and disease specific symptoms in long-term thyroid cancer survivors: a study from the population-based PROFILES registry. Acta Oncol..

[14] Haugen BR, Alexander EK, Bible KC (2016). 2015 American Thyroid Association management guidelines for adult patients with thyroid nodules and differentiated thyroid cancer: the American Thyroid Association Guidelines Task Force on Thyroid Nodules and Differentiated Thyroid Cancer. Thyroid..

[15] Links TP, Huysmans DA, Smit JW (2007). Guideline ‘differentiated thyroid carcinoma’, including diagnosis of thyroid nodules. Ned Tijdschr Geneeskd..

[16] Gort M, Otter R, Plukker JT, Broekhuis M, Klazinga NS (2010). Actionable indicators for short and long term outcomes in rectal cancer. Eur J Cancer..

[17] van der Sanden GA, Coebergh JW, Schouten LJ, Visser O, van Leeuwen FE. Cancer incidence in The Netherlands in 1989 and 1990: first results of the nationwide Netherlands Cancer Registry. Coordinating Committee for Regional Cancer Registries. *Eur J Cancer*. 1995;31A(11):1822–9. 10.1016/0959-8049(95)00355-m 8541107

[18] Bates MF, Lamas MR, Randle RW (2018). Back so soon? Is early recurrence of papillary thyroid cancer really just persistent disease?. Surgery..

[19] van Dijk D, Plukker JT, van der Horst-Schrivers AN (2011). The value of detectable thyroglobulin in patients with differentiated thyroid cancer after initial ^131^I therapy. Clin Endocrinol (Oxf)..

[20] de Groot JW, Links TP, Rouwe CW, van der Wal JE, Hofstra RM, Plukker JT (2006). Prophylactic thyroidectomy in children who are carriers of a multiple endocrine neoplasia type 2 mutation: description of 20 cases and recommendations based on the literature. Ned Tijdschr Geneeskd..

[21] Robbins KT, Medina JE, Wolfe GT, Levine PA, Sessions RB, Pruet CW. Standardizing neck dissection terminology. Official report of the academy’s committee for head and neck surgery and oncology. *Arch Otolaryngol Head Neck Surg*. 1991;117(6):601–5. 10.1001/archotol.1991.01870180037007 2036180

[22] Hayward NJ, Grodski S, Yeung M, Johnson WR, Serpell J (2013). Recurrent laryngeal nerve injury in thyroid surgery: a review. ANZ J Surg..

[23] Gourin CG, Tufano RP, Forastiere AA, Koch WM, Pawlik TM, Bristow RE (2010). Volume-based trends in thyroid surgery. Arch Otolaryngol Head Neck Surg..

[24] Stavrakis AI, Ituarte PH, Ko CY, Yeh MW. Surgeon volume as a predictor of outcomes in inpatient and outpatient endocrine surgery. *Surgery*. 2007;142(6):887–99 **(discussion 887–99)**. 10.1016/j.surg.2007.09.003 18063073

[25] Adam MA, Thomas S, Youngwirth L (2017). Is there a minimum number of thyroidectomies a surgeon should perform to optimize patient outcomes?. Ann Surg..

[26] Lifante JC, Duclos A, Couray-Targe S, Colin C, Peix JL, Schott AM (2009). Hospital volume influences the choice of operation for thyroid cancer. Br J Surg..

[27] Verhoef C, van de Weyer R, Schaapveld M, Bastiaannet E, Plukker JT (2007). Better survival in patients with esophageal cancer after surgical treatment in university hospitals: a plea for performance by surgical oncologists. Ann Surg Oncol..

[28] Verloop H, Louwerens M, Schoones JW, Kievit J, Smit JW, Dekkers OM (2012). Risk of hypothyroidism following hemithyroidectomy: systematic review and meta-analysis of prognostic studies. J Clin Endocrinol Metab..

[29] Su SY, Grodski S, Serpell JW (2009). Hypothyroidism following hemithyroidectomy: a retrospective review. Ann Surg..

[30] Edafe O, Antakia R, Laskar N, Uttley L, Balasubramanian SP (2014). Systematic review and meta-analysis of predictors of post-thyroidectomy hypocalcaemia. Br J Surg..

[31] Shindo ML, Caruana SM, Kandil E (2014). Management of invasive well-differentiated thyroid cancer: an american head and neck society consensus statement. AHNS consensus statement. Head Neck..

[32] Hay ID, Hutchinson ME, Gonzalez-Losada T, et al. Papillary thyroid microcarcinoma: a study of 900 cases observed in a 60-year period. *Surgery*. 2008;144(6):980–7 **(discussion 987–8)**. 10.1016/j.surg.2008.08.035 19041007

[33] Ito Y, Miyauchi A, Oda H (2018). Low-risk papillary microcarcinoma of the thyroid: a review of active surveillance trials. Eur J Surg Oncol..

[34] Oda H, Miyauchi A, Ito Y (2016). Incidences of unfavorable events in the management of low-risk papillary microcarcinoma of the thyroid by active surveillance versus immediate surgery. Thyroid..

[35] Miyauchi A, Ito Y, Oda H (2018). Insights into the management of papillary microcarcinoma of the thyroid. Thyroid..

[36] Links TP, de Heide LJ, Janssen M (2015). Guideline thyroid cancer including diagnostics of the nodule. Ned Tijdschr Geneeskd..

