# Supplementary material for: Surgical Complications and Referral Patterns in 567 Patients with Differentiated Thyroid Cancer in the Northern Region of the Netherlands: A Population-Based Study Towards Clinical Management Implementation
Source: Ann Surg Oncol. 2020 Apr 23;27(10):3872–81. doi: 10.1245/s10434-020-08470-1 (PMC7471192; doi:10.1245/s10434-020-08470-1)
Supplement: Supplementary file 1 — Supplementary material 1 (DOCX 13 kb) [file 10434_2020_8470_MOESM1_ESM.docx]

Supplementary Table 1. Demographics of in- and excluded patients.

|  | **N included patients (%)** | **N excluded**  **patients (%)** |
| --- | --- | --- |
| **Total** | **567 (100)** | **213 (100)** |
| Male  Female | 150 *(26.5)*  417 *(73.5)* | 61 *(28.6)*  152 *(71.4)* |
| Age at diagnosis (mean ±SD) | 49.2 (±17.7) | 56.4 *(±17.7)* |
| Papillary carcinoma  Follicular carcinoma (including Hürthle) | 419 *(73.9)*  148 *(26.1)* | 137 *(64.4)*  76 *(35.7)* |
| TX  T1  T2  T3  T4 | 20 *(3.5)*  139 *(24.5)*  213 *(37.6)*  112 *(19.8)*  83 *(14.6)* | 73 *(34.3)*  26 *(12.2)*  66 *(31.0)*  19 *(8.9)*  29 *(13.7)* |
| NX  N0  N1 | 319 *(56.3)*  89 *(15.7)*  159 *(28.0)* | 144 *(76.7)*  27 *(12.7)*  42 *(19.7)* |
| MX  M0  M1 | 165 *(29.1)*  376 *(66.3)*  26 *(4.6)* | 91 *(42.8)*  110 *(51.6)*  12 *(5.6)* |

Number (N) with percentages, unless stated otherwise. T: tumor; N: nodes; M: metastases; X: unknown.

Supplementary Table 2. Other complications

|  | Number |
| --- | --- |
| Postoperative bleeding | 33 |
| Asthma cardiale  Dysarrythmias (including AF)  Postoperative hypertension  Chest pain | 1  6  1  1 |
| Postoperative larynx edema | 5 |
| Per-operative defect trachea  Trachea-esophageal fistula | 2  2 |
| Hoarseness with intact recurrent laryngeal nerve  Hoarseness because of supraglottic hematoma  Hematoma vocal cord | 2  1  1 |
| Chyle leak | 7 |
| Horner syndrome (temporary)  Facial nerve palsy  Accessory nerve palsy  Hypoglossal nerve palsy (temporary)  Vagal nerve palsy | 2  2  4  2  1 |
| Wound infection (antibiotics or re-operation)  Postoperative seroma | 22  1 |
| Haematothorax | 1 |
| Upper respiratory infection  Pneumonia  Aspiration pneumonia  Respiratory insufficiency  Per-operative iatrogenic lesion lung | 1  9  1  1  1 |
| Deep venous thrombosis  Pulmonary embolism | 1  2 |
